# Enabling data-driven design of block copolymer self-assembly

**DOI:** 10.1038/s41597-025-05379-w

**Published:** 2025-06-21

**Authors:** Chiara Magosso, Irdi Murataj, Michele Perego, Gabriele Seguini, Debra J. Audus, Gianluca Milano, Federico Ferrarese Lupi

**Affiliations:** 1 Department of Electronics and Telecommunications, Politecnico di Torino, 10129 Turin, Italy; 2 Advanced Materials Metrology and Life Sciences Division, INRiM (Istituto Nazionale di Ricerca Metrologica), 10135 Turin, Italy; 3 CNR-IMM, Unit of Agrate Brianza, Via C. Olivetti 2, I-20864 Agrate Brianza, Italy; 4 Materials Science and Engineering Division, National Institute of Standards and Technology, Gaithersburg, Maryland 20899 USA

**Keywords:** Molecular self-assembly, Electronic devices, Electronics, photonics and device physics

## Abstract

Here we present a database composed of scanning electron microscope images of self-assembled block copolymers. The fabrication process parameters, structural properties and microscope information are all contained in the image metadata, making a group of images a database on its own. This approach has numerous advantages including ease of sharing, reusability of information and resilience against user errors. This database follows the digital International System of Units principles and is complemented by a graphical user interface for process metadata insertion and an automated algorithm for image analysis to retrieve structural properties of the nanostructures. Databases such as this one, together with data-driven approaches, enable users to rationally design new materials with the desired properties by understanding the relationship between fabrication parameters and material structure. The here reported database, that contains around 1747 images of lamellar phase and lying down cylinders self-assembled block copolymers along with associated metadata, is structured so it can be continuously expanded by the research community including also samples with different block copolymers morphologies.

## Background & Summary

Block copolymers (BCPs) are macromolecules composed of two or more chemically distinct and, in our case, immiscible homopolymers joined together by a covalent bond. When in the melt state and below order-disorder transition temperature, BCPs can phase separate and self-assemble, enabling the formation of microstructures (such as spheres, cylinders, gyroids, and lamellae) with characteristic dimensions typically on the scale of a few tens of nanometers^1^. Thanks to these distinctive structures, BCPs have found broad application^2^, including microelectronics^3,4^, nanophotonics^5–7^, sensing^8–10^, and drug delivery^11^.

In order to accelerate the use of these nanoscale materials, approaches such as the Materials Genome Initiative^12^ (MGI) have been developed. The overall goal of the MGI is to provide the means to reduce the cost and time of materials discovery, optimization, and distribution by exploiting the synergy between data, computational tools and experiments. A key aspect of this approach is the generation of databases to build computational models and artificial intelligence tools for extracting values from data to rationally design new materials. However, the generation of such databases in the field of material science and nanotechnology is challenging. One approach is to use data from the literature. However, this data is frequently unstructured, lacking standardization, and usually missing essential metadata, thus hindering the real extraction of values from these databases^13^. The second approach is to generate a database directly from experiments or simulations. While this approach can circumvent issues such as missing metadata and lack of standardization, it is costly in terms of materials, time, and expense. For BCPs, there have been a handful of data efforts over the years primarily focused on the former approach of literature extraction. This includes the “Thermodynamics of Polymer Blends”^14^ in the *Physical Properties of Polymers Handbook*^15^ and the Polymer Property Predictor and Database^16,17^, a catalog of Flory-Huggins $\chi$ parameters. In addition to extracting the $\chi$ parameter from literature, another approach is to estimate the $\chi$ parameter using solubility parameters, which can be computed via group contribution methods^18–21^. However, this method is prone to introducing additional errors^14^. The $\chi$ parameter together with self-consistent field theory (SCFT) can be used to predict both the microstructure and the characteristic domain size of both block copolymers and blends of homopolymers and block copolymers^22^. While broadly used^2^, SCFT is only semi-quantitative in part due to its mean-field assumption^23^. Due to these issues, Rebello *et al*. created the Block Copolymer Phase Behavior Database^24,25^ (BCDB) based directly on experimental identification of BCP microstructures (e.g., lamellae, hexagonal packed cylinders). In their data resource, they cataloged BCP chemistries, block fractions, and the resulting microstructures. To demonstrate the usefulness of such databases, in another work the database is coupled with machine learning to predict the microstructure^26^.

**Fig. 1:**
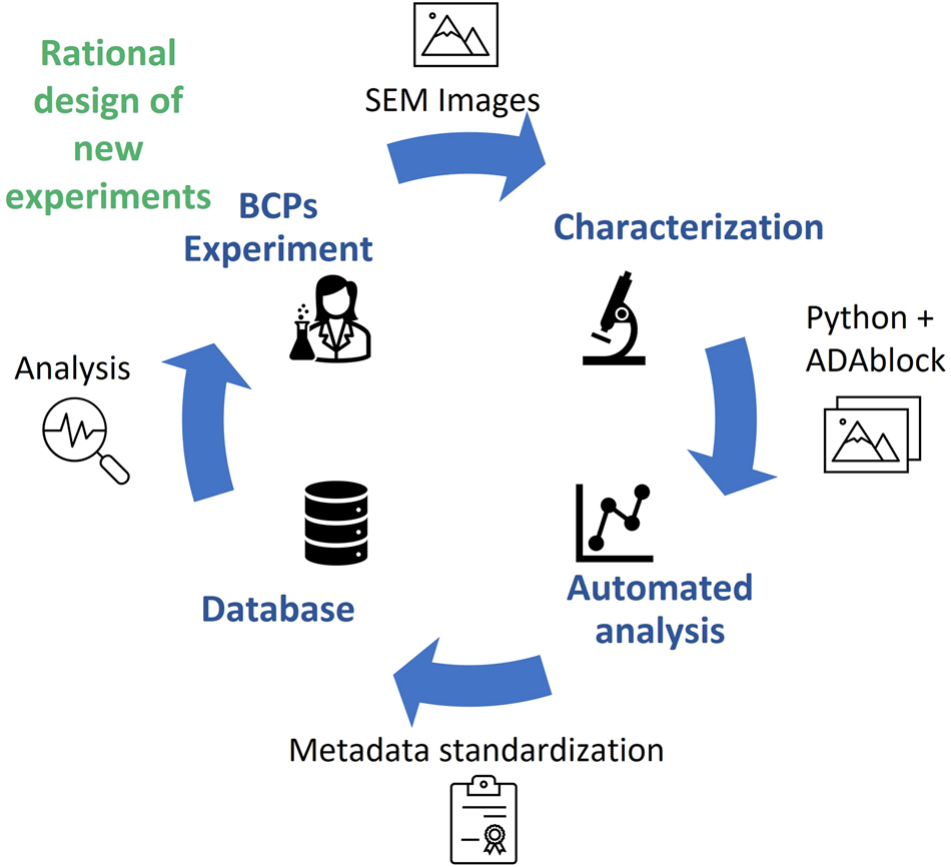
Workflow of database creation. The BCP self-assembly was performed at different experimental conditions. The samples were afterwards characterized via SEM imaging. Subsequent automated image analysis was conducted using Python and ImageJ, with metadata insertion performed using dedicated software to ensure data standardization. The resulting database was created in two formats: each image is accompanied by its own metadata, and a comma-separated values (CSV) file containing the metadata of the entire database. Both forms of the database can be used for aggregate data analysis and consequently rationally design new experiments^12^.

Despite these advances, a full understanding of the effect of processing parameters, especially on quantities such as the size of the domains, is still lacking. In this context, our database aims to tackle limitations of previous approaches. Specifically, our effort is focused on self-assembling BCPs and our goal, boosted by the support of the reference community, is to extend the database presented here to other laboratories, characterization techniques and structures, ultimately facilitating the establishment of a standardized reference database of BCPs structures related to their fabrication process. As shown in Fig. 1, the database is currently a collection of SEM (scanning electron microscope) micrographs of lamellar and lying down cylinders phase-separated BCPs that were fabricated under a wide variety of processing conditions. These structures were fabricated and analyzed over many years at the Italian National Institute of Metrological Research (INRiM) and Italian National Research Council - Institute for Microelectronics and Microsystems (CNR-IMM) laboratories. Micrographs of these nanostructures, which are stored as Tagged Image File Format (TIFF) images, have been analyzed using custom-built automated software to enhance reproducibility by eliminating human bias in the analysis of structural properties of BCPs. The result of such structural analysis and the fabrication parameters are inserted into the micrographs metadata following the digital International System of Units (D-SI) principles^27^. In this way a collection of images becomes itself a database that can be exploited as a base for the development of a materials-by-design^28^ approach. The data-driven design approach, indeed, can replace the trial-and-error approach commonly used for this type of sample, allowing for fewer experiments to be performed to obtain a new material or sample as shown in Fig. 1. Furthermore, we have designed our database and related methods such that it can easily be dynamically expanded to include additional data coming from the community.

## Methods

### Sample preparation

BCP samples are generated through a multi-step fabrication process. Here, we will highlight the most significant steps, but additional details can be found in previous works^29,30^. First, a substrate is chosen, which is typically but not always^31^ a silicon wafer. Second, a random copolymer (RCP) is deposited via spin coating and annealed to induce grafting of the RCP to the substrate. The purpose of the RCP is to promote self-assembly of BCPs in a predefined direction (e.g., perpendicular ordering of lamelle BCP nanostructures^32^). Third, a BCP is deposited via spin coating and annealed to enable the self-assembly. For each of these fabrication steps there are multiple parameters that can be tuned, each of which influences the resulting BCP morphology. The most relevant materials and processing parameters have been recorded in the database and defined in a separate markdown file. Concerning the involved materials, parameters include but are not limited to the copolymer chemical composition, number average molecular mass, dispersity, solvent and thickness of the layer, which may be tuned separately from number average molecular mass^32^. Instead, processing parameters include the temperature, time, and thermal ramp of the thermal treatment. Furthermore, the BCP layer can be as is or blended with homopolymers (we refer to the first case as neat, while the second as blend). For the blend case, homopolymer parameters are added in the database including the chemical identity, number average molecular mass and weight fraction. A complete list of parameters can be found in a markdown file^33^.

Currently, most samples in the database are composed of poly(styrene-*block*-methylmethacrylate) (PS-*b*-PMMA) with a volume fraction of around 0.5, consistent with the lamellar morphology^34^. These BCPs were purchased from Polymer Source Inc (sample numbers: P3964-SMMA, P1573-SMMA, P5646-SMMA, P10197-SMMA and P5539-SMMA). The homopolymers used were synthesized according to F. Ferrarese Lupi *et al*.^29^ To demonstrate the flexibility of the database, we also included some samples of polystyrene-*block*-poly(dimethylsiloxane-*random*-vinylmethylsiloxane) (PS-*b*-P(DMS-*r*-VMS)) with a volume fraction of around 0.7, consistent with the lying down cylinder morphology^34^. These BCPs were the same as in Giammaria, T. J. *et al*.^35^. This choice allows us to take advantage of the fact that lying down cylinders result in fingerprint-like patterns similar to lamellae, and the final morphology appears analogous when analyzed through SEM imaging.

### Image acquisition

**Fig. 2:**
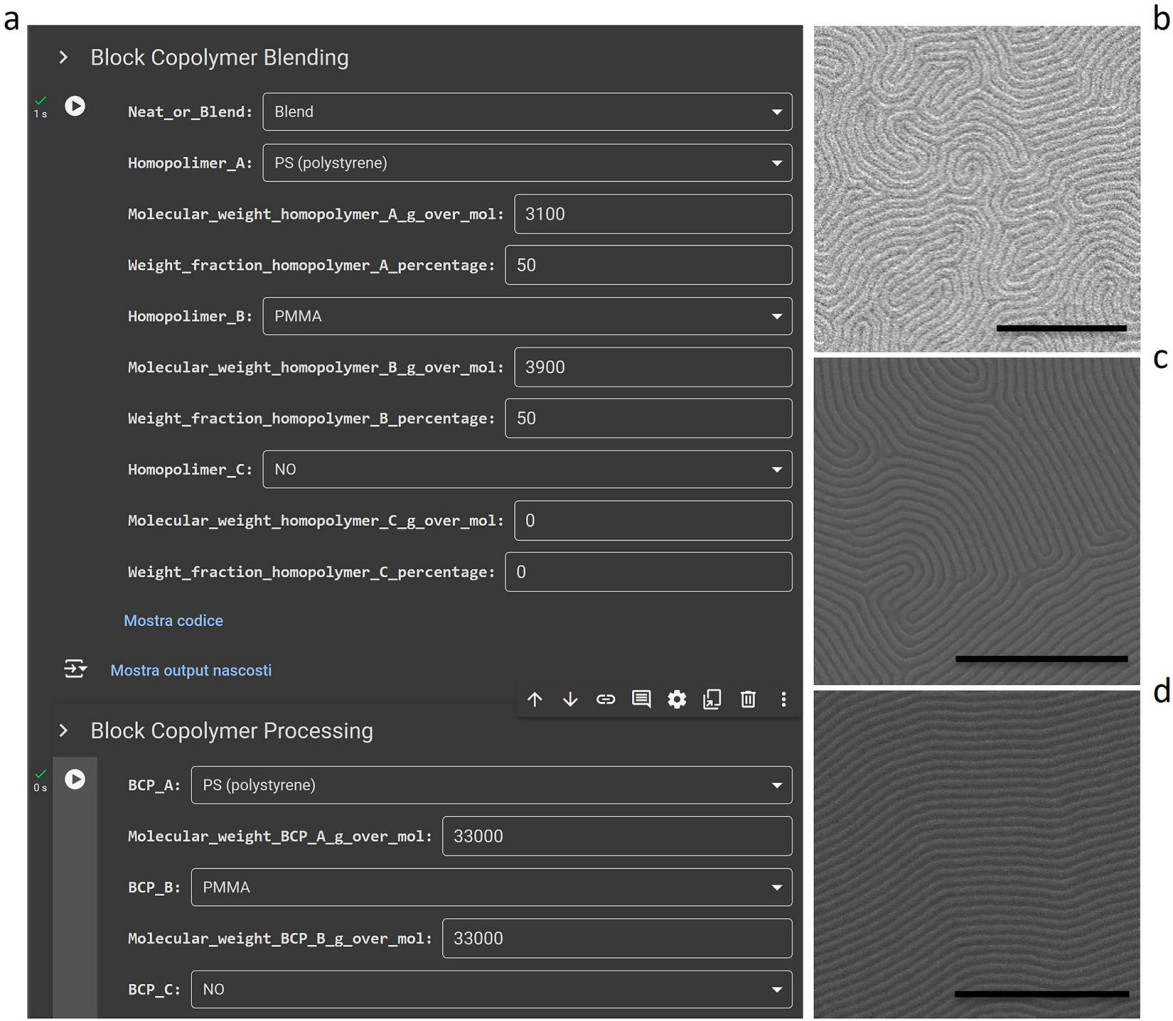
Metadata insertion tool. (**a**) Application developed to insert process parameters into SEM images metadata in a standardized way between different laboratories by fixing unit of measures and nomenclature. (**b**–**d**) Example SEM images (scale bar 500 nm).

To unveil the self-assembled nanopattern, we first selectively remove one of the two chemistries of the BCP to ensure enough contrast for SEM images acquisition. The sample pitch is not affected by this procedure because it is a measure of periodicity^36^. Example images are shown in Fig. 2b–d. Images are acquired from different SEMs and at different SEM settings including brightness, contrast, and magnification. Furthermore, the database also includes multiple images taken from different areas of the same sample and at different SEM magnifications, as well as includes images of different samples made with the same experimental parameters, allowing to analyze both intra-sample variability and sample-to-sample variability.

### Process metadata insertion

To ensure standardized insertion of process parameters into SEM micrographs metadata, especially for samples fabricated and analyzed in different laboratories and/or by different operators, we developed a metadata insertion graphical user interface (GUI), depicted in Fig. 2a. The GUI prompts the user to first upload the image (e.g., Fig. 2b–d) into which the metadata will be inserted, and then provides distinct sections for the various layers of the sample. The GUI is available on GitHub^37,38^ for users to allow them to add their own metadata into their SEM images. The GUI has already been generalized to accommodate its use with different BCP morphologies. However, we ask users to contact us if additional fields (e.g., other processing parameter) or additional values (e.g., a new substrate or solvent) are required, in order to maintain a controlled vocabulary. Similarly, we ask users to contact us for uploading their samples. In the future, this process may become automated.

Within the GUI, the user is guided to input process parameters. For numerical quantities such as number average molecular mass, the user is prompted to enter the value, while for string-based parameters such as polymer chemistry, the user has to make a choice based on a drop-down menu to ensure consistent nomenclature. Moreover, the program pre-defines units of measure for the parameters to maintain standardization. These indicated units correspond to the typical ones used in the laboratory for these quantities, further ensuring consistency.

### Automated image analysis and structural metadata insertion

Before insertion in the database, the acquired SEM images are analyzed to determine a variety of structural quantities such as sample pitch (domain spacing) of the BCP or details about defects (deviations such as brakes or junctions from perfectly parallel straight lines). To reduce bias and error while making the image analysis efficient and faster, especially when handling large datasets, we developed an automated image analysis software, which can be accessed at GitHub^37–39^. Specifically, this program automatically analyzes lamellae and lamellae-like structure (e.g. lying down cylinders) images and adds the extracted structural quantities directly to the SEM image. The work presented here, both the analysis approach and the database itself, can be generalized. For all the other BCPs morphologies such as cylinders and spheres a suitable algorithm needs to be developed to properly acquire the characteristic feature of the different self assembled structures from SEM images. Our database is also open to other characterization techniques (different to the SEM one) such as atomic force microscopy and grazing-incidence small-angle X-ray scattering (GISAXS). However, additional code needs to be developed to actually integrate these characterization techniques in the automated algorithm.

**Fig. 3:**
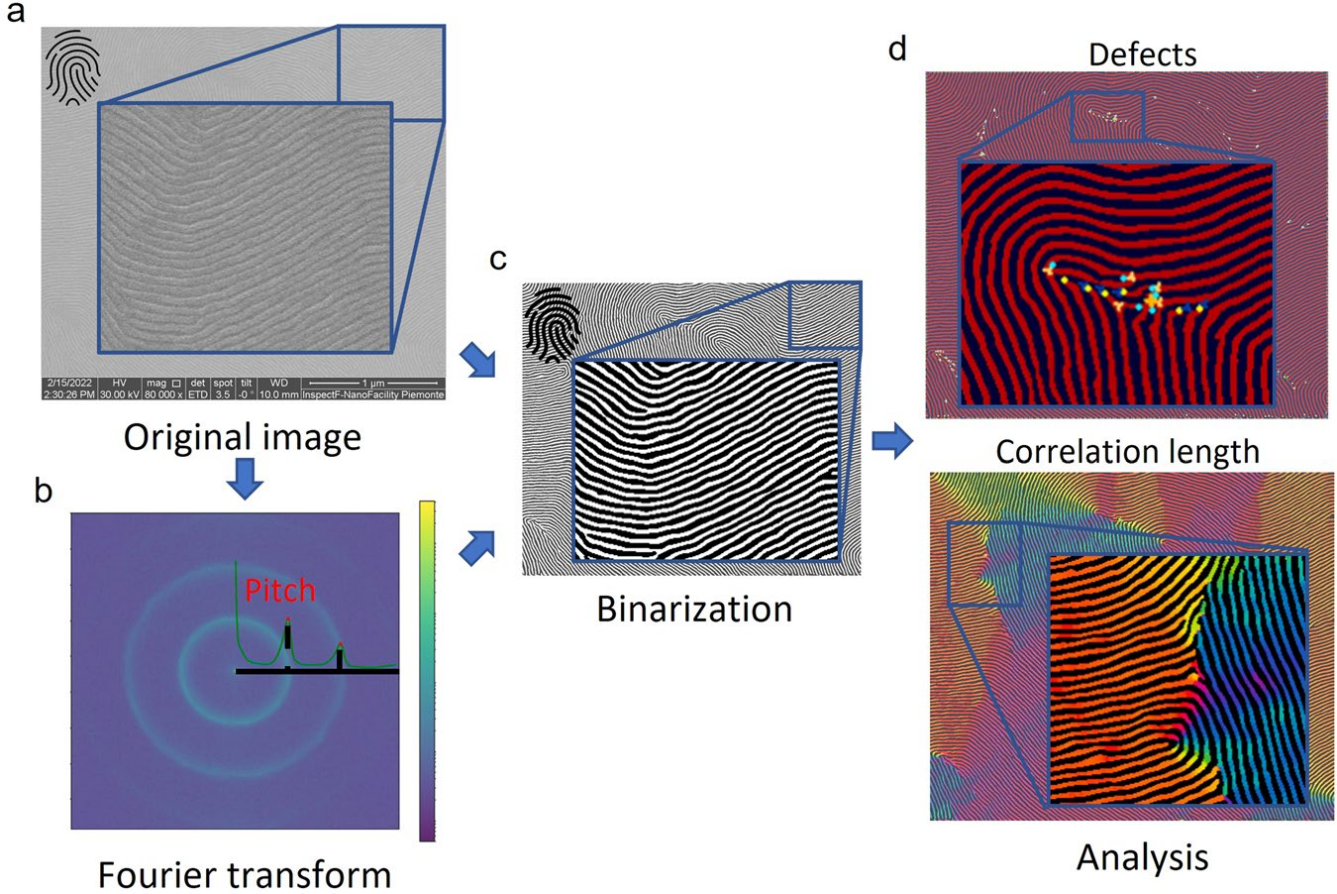
Automated image analysis process flow. After reading the process parameters from the SEM image, we crop the scale bar (**a**) and perform a fast Fourier transform (**b**) in order to obtain the sample pitch (*l*_0_) value. Having sample pitch value and resolution, the Fingerprint-Enhancement-Python package^40,41^ is used to binarize the pattern (**c**); the last step is to use the modified ADAblock^39^ macro to obtain the structural parameters (**d**). The automated analysis ends with the insertion of the extracted parameters into the image metadata.

The program developed in Python is graphically summarized in Fig. 3 and works as follows. Initially, the image (Fig. 3a) is cropped to remove the SEM data bar, and both the process metadata previously inserted and the SEM image metadata are read. Next, the sample pitch (*l*_*0*_) is determined via a Fast Fourier Transform (*FFT*). To enhance the reliability of the technique, a radial mean of the *FFT* is performed (Fig. 3b) and then smoothed using a spline function. The location of the first peak is then transformed from Fourier space to real space in order to yield *l*_*0*_. We also estimate the uncertainty of the calculated *l*_*0*_ by considering the peak full-width half maximum, which is then converted to a standard deviation by assuming a Gaussian shape for the peak.

Additional structural quantities including correlation length (average distance over which the orientational order of the film is preserved) and defect types and locations also need to be extracted in order to properly describe the sample morphology. To determine these quantities, the cropped SEM image is binarized (Fig. 3c) following the work of Murataj *et al*.^31^ using a function of the Fingerprint-Enhancement-Python package^40,41^. The advantage of this algorithm is that it allows binarization of SEM images even from poor SEM quality and with a reduced introduction of spurious defects with respect to conventional binarization techniques. Next, the program executes an ImageJ macro called ADAblock^42,43^, which has been modified^39^ to align with the automated analysis process flow. Specifically, the ADAblock GUI was removed and preprocessing steps such as cropping and binarization were removed as the preprocessing is already handled outside of ADAblock in the main Python program. We exploited ADAblock for analyzing the lamellar and lamellar-like structures from the binarized image, providing valuable information (Fig. 3d) including but not limited to correlation length, defect identification and defect density (number of defects or defect pairs per unit area, depending on the defect type as specified in ref. ^42^). For a complete list of all the parameters analyzed by automated image analysis software refer to the markdown file^33^.

The results of the analysis of the lamellae structure are passed to the main Python program that incorporates them as image metadata. Upon completion of the analysis, the user is presented with an image containing all metadata required to experimentally reproduce and describe the sample. This includes fabrication process parameters, SEM image parameters, and structural analysis data from the automated analysis. If the automated image analysis software encounters an error such as inability to binarize the image, the image is not included in the database.

## Data Records

The database^33^ presented here is an extensive effort as it features BCP experiments performed in different laboratories with different instruments. As previously discussed it is composed of SEM images in TIFF format containing embedded standardized metadata for all the different aspects of the sample (fabrication, characterization, structural analysis).

Within the image metadata, the fabrication process data are located under the ‘ImageDescription’ tag in JavaScript Object Notation (JSON) format, and the structural analysis data are located under the ‘Software’ tag in JSON format. The complete imaging metadata depends on the specific SEM instrument and are left as originally inserted by the instrument even if some of the main values have been extracted and reported among the structural parameters. The images that make up the database, presented in this work are stored in a Zenodo repository^33^ along with a single CSV file that is generated by the automated image analysis algorithm. Each row of the CSV corresponds to a particular image and each column corresponds to particular metadata. The CSV file therefore, summarizes all the key metadata embedded in the images with some additional information on the data cleaning as detailed in the technical validation section and a classification in systems based on copolymer chemical composition as detailed further below. In the Zenodo repository, there is also a markdown (md) file that defines all of the nomenclature used in the image metadata and in the CSV.

The database presented in this work has been structured so it can be continuously updated with new samples as described in the Methods section, while this Data Descriptor details its current content. To demonstrate the ability to handle heterogeneous data from different sources and different material systems we have included both data from prior works^29,35,44–48^, and new samples purposely fabricated for this work.

**Fig. 4:**
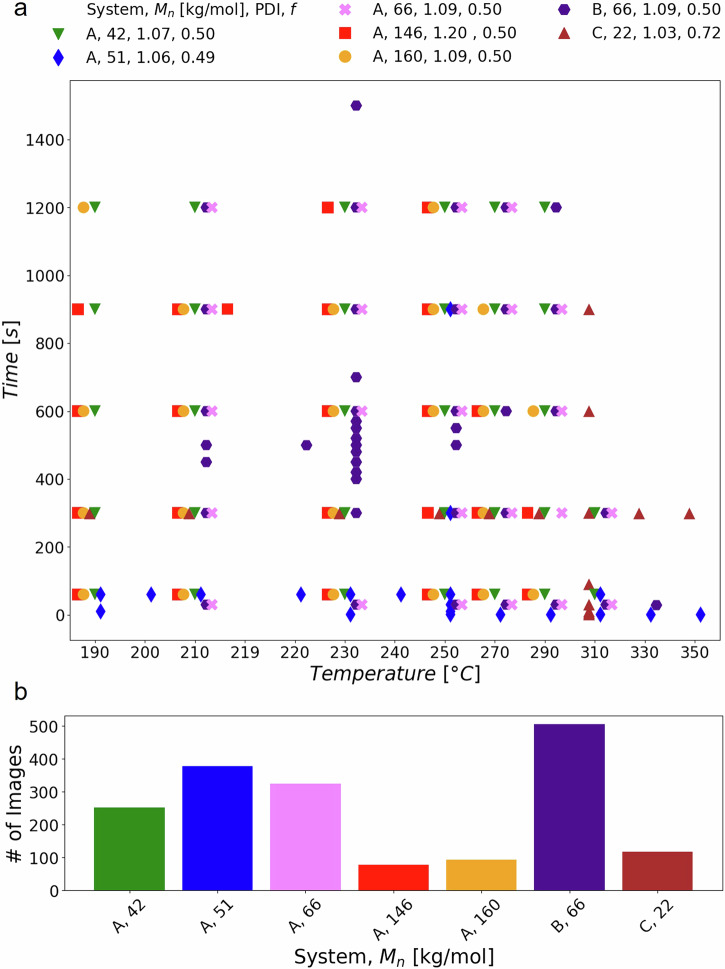
Categorical graph reporting the process condition of data currently present in the database and number of images per system. (**a**) Every point represents one or more samples produced in that particular condition, described by number average molecular mass (*M*_*n*_), temperature and time. Every sample was characterized by one or more SEM images taken on different areas of the sample. Note that the temperature values are categorical and an offset across the various systems is added in the x-axis to ensure the graph readability. The legend is structured as follows: the first element indicates the System. A corresponds to PS-*b*-PMMA, Neat, both samples from prior work^44–48^ and new samples; B corresponds to PS-*b*-PMMA, Blend^29^ with PS homopolymer (*M*_*n*_ = 3.1 kg/mol) and PMMA homopolymer (*M*_*n*_ = 3.9 kg/mol); C corresponds to PS-*b*-P(DMS-*r*-VMS)^35^, Neat. The second element indicates the number average molecular mass in [kg/mol], the third is the dispersity (PDI) while the fourth is the volume fraction of one block (*f*) that was determined from the number average molecular mass. (**b**) The bar height represents the number of images taken for each system divided by System and number average molecular mass in [kg/mol]. A total of 1747 images and 180 unique fabrication process conditions are included in the plots.

The database currently contains 1747 images of samples made and characterized in two different laboratories (INRiM and CNR), which together span 180 unique fabrication process conditions, including different chemical composition of polymers (system A corresponds to PS-*b*-PMMA, Neat; system B corresponds to PS-*b*-PMMA, Blend with PS homopolymer and PMMA homopolymer; system C corresponds to PS-*b*-P(DMS-*r*-VMS), Neat), polymers with multiple number average molecular masses, different processing times, and different processing temperatures as shown in Fig. 4. Each point corresponds to a condition from which there is at least one sample demonstrating the breadth of the processing conditions covered by the database. The SEM images of samples at a given condition (number average molecular mass, temperature and time) are both from different samples and from the same sample at different areas. Furthermore, the images were taken with different SEMs, at different magnifications, at different brightnesses, and contrasts.

## Technical Validation

### Aggregate data analysis

To ensure accurate results of our automated analysis, we compared the sample pitch for each image before and after the binarization of the micrograph. A 5% acceptance is allowed on the relative uncertainty. Any image with relative uncertainty higher than 5% is considered as failing the data cleaning is marked ‘True’ in the CSV file under the binarization check column. This approach is exploited to automatically validate the binarization process, since some SEM images can be wrongly binarized for various reasons, for example because the starting image is low quality (blurred, inadequate resolution, insufficient contrast between the polymer phases and so on). Individual SEM images with metadata are left undisturbed in case different criteria are desired in the future. After the described data cleaning, the dataset is composed of 1462 images of samples which span 177 unique fabrication process conditions across the three different systems, multiple number average molecular masses, different processing times, and different processing temperatures.

**Fig. 5:**
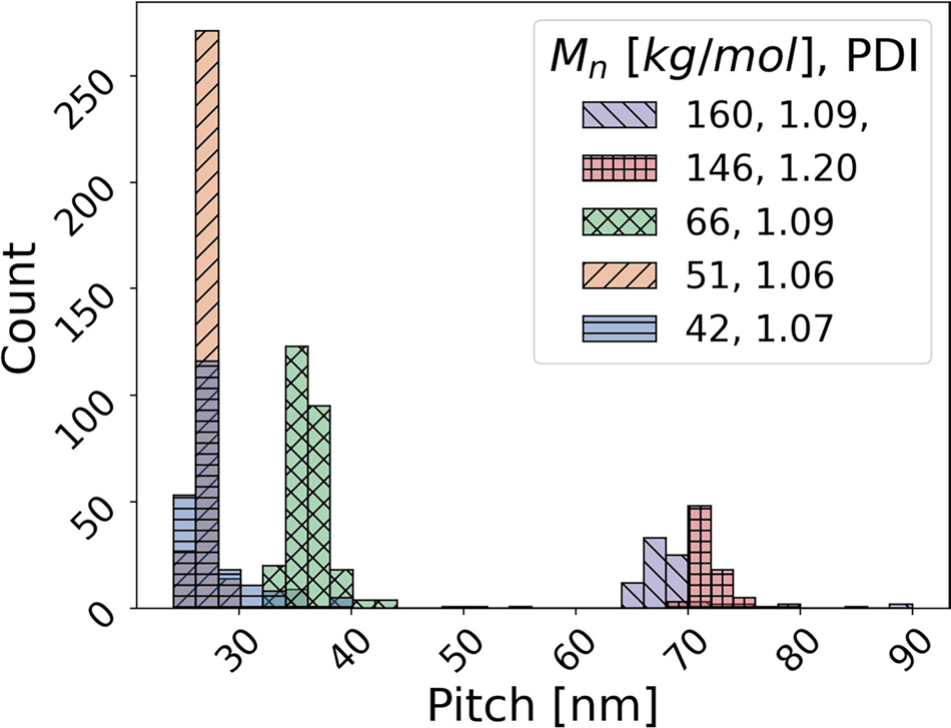
Histogram of sample pitch categorized by number average molecular mass. A total of 962 images are included in the plot.

To further validate our data, we analyze several trends. First, we used the cleaned data of the most common BCP chemistry, PS-*b*-PMMA for the neat case only (the System A data represented in Fig. 4). In Fig. 5 the histogram of sample pitch is plotted based on the average molecular mass, since this is the parameter with the greatest contribution^49^. The modes of the sample pitch histograms are approximately (68, 72, 36, 26, and 26) nm for number average molecular masses of (160, 146, 66, 51, and 42) kg/mol, respectively. The obtained results are in accordance with literature results^50^. It is known^30,51^ that the sample pitch value is mainly dependent on the molecular mass and as can be seen from Fig. 5 the sample pitch mode decreases with the number average molecular mass. However, this trend is disrupted by samples with large dispersities^52^, such as the 146 kg/mol sample, which has a dispersity of 1.2, unlike the other samples with values close to unity.

**Fig. 6:**
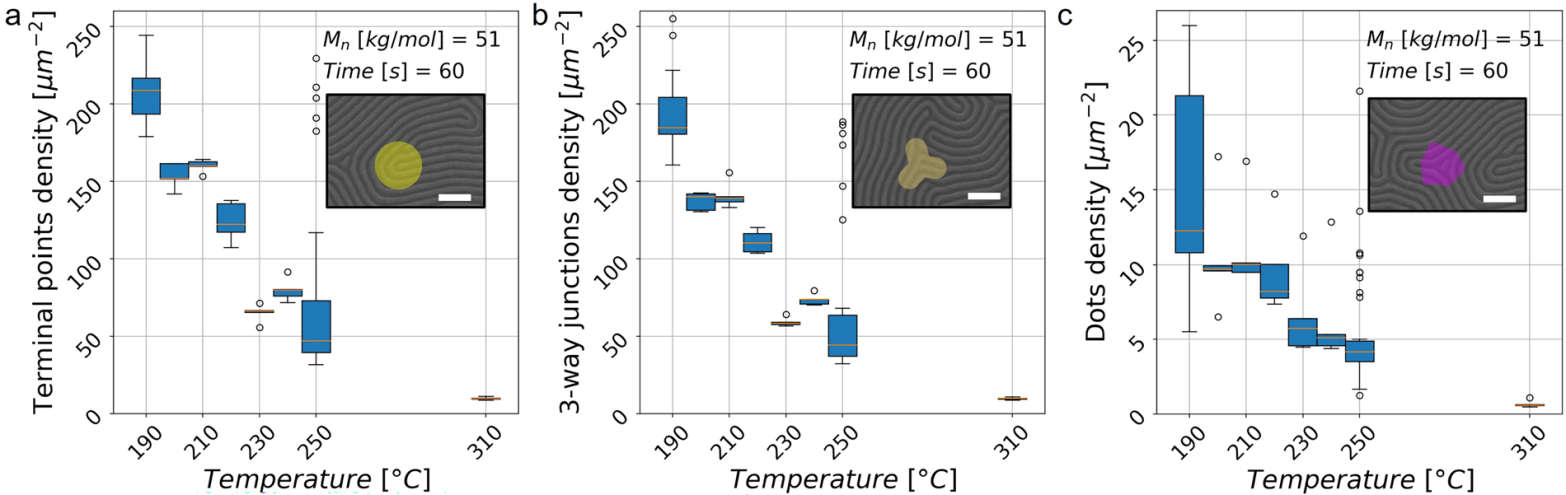
Defect density as a function of processing temperature. (**a**–**c**) By fixing for system A the number average molecular mass at 51 kg/mol and time at 60 s, we study the trend of different types of defect density as the temperature varies. SEM images (scale bars are 100 nm) with a detail of the different defect types and associated ADAblock^42^ markers are reported as insets. (**a**) Box and whisker plot of terminal points defect pair density. (**b**) Box and whisker plot of 3-way junction defect pair density. (**c**) Box and whisker plot of dot defect density. Orange lines represent the median, boxes represent the 25th to 75th percentile, whiskers are 1.5 IQR and empty circles are outliers. A total of 92 images are included in all of the plots.

Second, we look at the defect types as classified by ADAblock^42^. For a two dimensional line pattern such as our data, defects are first classified as junctions, terminal points and dots (see also Fig. 6 in ref. ^42^). This classification is determined through an analysis of defect pixels and their neighbors (see also Fig. 7 in ref. ^42^). For our system, the defect densities for three types of defects (terminal points, 3-way junctions, and dots) at a fixed number average molecular mass and processing time are shown in Fig. 6. The graphs indicate that the defect densities decrease with increasing process temperature, consistent with previous findings in the literature^53^. Additionally, considering the criteria adopted by ADAblok for the evaluation of defect density and by considering both defects from positive and negative phases, we find that terminals and junctions defect pair density types (Fig. 6a,b) are significantly more common than dots defect density type (Fig. 6c). Together these two analyses validate our database.

### Metrological traceable validation

**Fig. 7:**
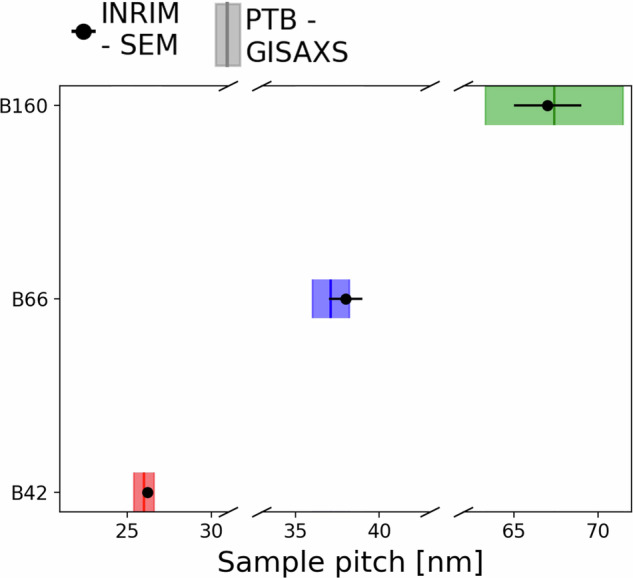
Interlaboratory comparison. We study the sample pitch value of three different samples through two different characterization techniques. The dots represent the SEM measurements of the weighted average pitch where the uncertainty is standard error of mean determined via a weighted average of the pitch using the measured standard deviations as described in the methods section. The boxes represent the dispersions of the sample pitches measured by GISAXS where the uncertainty contributions can be found in the work of Murataj *et al*.^50^, the measurement and uncertainty are estimated according to the combined standard uncertainty described in the work of Wernecke *et al*.^54^.

In addition to the aggregate data analysis on the dataset and the comparison with literature, we also performed on a subset of samples an interlaboratory comparison between the sample pitch determined from the automated analysis of our SEM images at INRiM and the one determined from GISAXS, a more resource intensive but metrologically traceable method, at PTB (Physikalisch-Technische Bundesanstalt). The GISAXS traceability to the SI^54^ ensures that the measurement results can be related to a reference standard through a documented, unbroken chain of calibrations. For this reason, GISAXS results can be considered as a metrological benchmark for validation. To do this comparison the sample patterns, fabricated as detailed in the methods section and in Refs. ^31,50^, are then transferred on the BCP substrate with an engraving technique^31^. Notably, the periodicity of the sample structure is not affected by this procedure^36^. Pattern transfer onto a stable substrate makes the samples more durable and prevents X-ray beam damage and degradation of the polymeric matrix during GISAXS and SEM measurements. These same samples are part of a larger interlaboratory comparison in the framework of a Versailles Project on Advanced Materials and Standards (VAMAS) Strategic Activity (TWA0/Project 3)^55^. A comparison of the sample pitch measurement results for three different samples obtained through SEM imaging and GISAXS results are shown in Fig. 7. The sample names B42, B66 and B160 reported in Fig. 7 indicate the corresponding neat block copolymer number average molecular mass of 42, 66 and 160 kg/mol while the self-assembly annealing process is performed at 230 °C for 600 s for all the three samples. We find that the values from our automated analysis of SEM images are within the confidence intervals of those measured via GISAXS, thus validating the SEM technique, the automated analysis, and the database. Being able to benchmark our SEM measurements with GISAXS is important not only because it validates our work with a measurement that is directly traceable to the SI, but also because it allows us to place our trust in a much more accessible technique (SEM) in terms of costs, ease of measurement and availability.

### A FAIR database

Finally, the database presented in this work follows the Findable, Accessible, Interoperable, and Reusable (FAIR) guiding principles as outlined by Wilkinson *et al*.^56^ Specifically, hereunder we analyze each aspect required for the database to be FAIR using letters and numbers such as F1 corresponding to those in Box 2 of their paper^56^.

The (meta)data is assigned with a Zenodo^33^ Digital Object Identifier (DOI) (F1). The metadata contains fabrication process parameters, structural parameters, and imaging parameters (F2, R1) using a machine-readable schema. Since each image has its own metadata, it is straightforward to associate the metadata to the corresponding data (F3). Together with the (meta)data we supply a CSV file reporting all the metadata contained in the different images along with the image names to make the search of a precise datapoint easier to the user (F4).

The data are publicly available using their DOI (A1) and free to download under Creative Commons Attribution 4.0 International (CC BY 4.0) license conditions (R1.1). The metadata are embedded in the data, and with the use of Python scripts, are readable to humans and machines (A1.1). However, having metadata embedded into data does not allow access to metadata if data are no longer available. For this reason the CSV file reporting the image metadata is also provided as part of the Zenodo repository (A2). Zenodo was specifically chosen as it is a trustworthy repository compliant to the guidelines on FAIR Data Management established by the European Commission states^57^. There is no need for authentication and authorization procedure since the goal of our database is to be open to the public (A1.2).

The metadata are in JSON format so they are readable from both machines and humans, they contain community agreed nomenclature (I2) and the data are reported in SI units by following D-SI recommendation or units recognized for use with the SI. Thus, they meet the domain-relevant community standards (R1.3). Data are images in TIFF format (I1). Our database is complete as is and is not based on other databases therefore qualified references to other (meta)data are non-applicable (I3).

As previously discussed, process parameters were added to the metadata in addition to the native image parameters ensuring sample provenience (R1.2).

## Data Availability

The codes can be accessed on GitHub at https://github.com/ChiaraMagosso/BlockMetrology^38^ & https://github.com/ChiaraMagosso/ADAblock/blob/ijMacro_updates_only/ADAblock.ijm^39^. A version release of the Python code (BlockMetrology) exploited in our work can be found at 10.5281/zenodo.15430176^37^. The database is available on Zenodo at 10.5281/zenodo.13927939^33^. All other data are available from the authors.
